# A Scientometric Analysis of Africa’s Health Science Journals Indexed in International and Regional Databases: A Comparative Analysis

**DOI:** 10.3389/ijph.2023.1606415

**Published:** 2024-01-15

**Authors:** Apatsa Selemani, Kondwani Wella, Yen-Fu Chen, Marta Vicente-Crespo, Olalekan Uthman, Jude Igumbor

**Affiliations:** ^1^ KUHES Libraries, Kamuzu University of Health Sciences, Blantyre, Malawi; ^2^ School of Public Health, Faculty of Health Sciences, University of the Witwatersrand, Johannesburg, South Africa; ^3^ Medical School, Warwick University, Coventry, United Kingdom; ^4^ African Population and Health Research Center (APHRC), Nairobi, Kenya

**Keywords:** research impact, African journals, scholarly communication, research evaluation, research metrics

## Abstract

**Objectives:** This study aimed to compare the geographic coverage, citation impact, subject trends and authorship collaboration pattern of African health science journals indexed in international and regional databases.

**Methods:** Data was collected from Ulrichs web serials directory, Web of Science (WoS), Scopus, PubMed, Google scholar, African Index Medicus (AIM) and African Journals Online (AJOL) between February 2023 and May 2023. Data was analysed using summary descriptive statistics such as percentages and interquartile ranges, and through network visualisation.

**Results:** More than 40 African countries had no any health science journal indexed in WoS, whereas 20 African countries did not have any health science journal indexed in AJOL and AIM. The Journal of Advanced research was the top performing journal on almost all journal metric lists such as Google scholar’s H5-Index, SNIP, Journal Impact Factor, and Citescore, except Journal Citation indicator.

**Conclusion:** The coverage of African health science journals by international citation databases is still limited which result in low scientific impact of many African health science journals. Authorship collaboration is related to historical ties among countries.

## Introduction

A number of studies indicate that, compared to other parts of the world, African health sciences research has had little scientific impact [1, 2]. This is mostly because there are few African journals included in the international citation databases [1, 3, 4], which reduces the visibility of the continent’s health science research. Several studies have reported an increase in scientific impact following indexing of journals in international and regional citation databases as it increases visibility [5–8]. Likewise, indexing status of a journal is one of the factors that influences author’s selection of journal to publish with. Hence, authors from Africa prefer publishing in journals from global north whose majority are indexed in popular international indexers.

However, this practice limits impact of local journals. Some authors also observed that it disadvantages local research as authors are forced to conduct research required by the global north journals [9, 10]. Additionally, international topical coverage is said to hinder growth of new fields of study and localisation of research as researcher’s are forced to focus on global issues at the expense of addressing local challenges [9, 11].

Scientometric analysis is the study of measuring and analysing science using quantitative approaches [12]. It is similar with bibliometrics, but differ on that bibliometric analysis focuses on quantitative analysis of bibliographic materials such as books, articles and conference papers. Scientometrics is one way of understanding scientific research activity, impact, collaboration and funding through proxies of publications, citation patterns, co-authorship and funding activity. The objects of measurement include scientific research articles, scientific journals, science authors, institutions, and countries [13]. This paper focuses on scientific journal’s coverage and impact, and scientific research collaboration by country. The journal metrics selected include Journal Impact Factor (JIF) and Journal Citation Indicator (JCI) by Web of Science (WoS), Source Normalised Impact per Paper (SNIP) and Citescore by Scopus, Hirsch Index (H5-Index) by Google scholar. This scientometric analysis followed the structure shown in Supplementary Figure S1.

These citation-based journal metrics have become a popular way to judge or evaluate journals and research published in them. They have gained acceptance and authority to the point where they are influencing the course of research [14]. They are used for evaluating research, hiring academic staff, making decisions about promotions and tenure, allocating funding for health research, ranking universities, choosing which journals to publish in, and informing readers or decision-makers about where to find evidence for reviews or evidence synthesis [14–16]. These choices unquestionably influence the production and direction of health research, and some have a direct impact on health interventions and policy.

One major issue that underlies many of these inherent challenges is the data source used to calculate metrics. For instance, some studies have reported that despite being a multidisciplinary database, Web of Science and Scopus under-represent some disciplines in their coverage, such as humanities and social sciences [17]. Whereas PubMed is a discipline-specific database focusing on biomedical literature hence may be more appropriate to be consulted as the data source for biomedical research evaluation.

There is limited evidence that compares scientific impact and research landscape of African journals based on their indexing status. Studies done previously either used one data source such as WoS or AJOL only or compared only two data sources [18, 19]. The main objective of this study was to compare the scientific impact of Africa’s health science journals indexed in international citation databases and regional databases.

## Methods

### Selection of Journals

We used web scraping, a data mining technique, to extract a list of African medical and health Sciences journals from Ulrichsweb global directory of serials. Ulrichsweb is one of the largest and key serials directories in the world and provides data with the level of detail required in this study [20]. We selected journals whose status was indicated as active. Ulrichsweb categorizes health related journals under medical and health science journals. We then manually searched WoS, Scopus, PubMed, African Journals Online (AJOL), African Index Medicus (AIM) for additional health science journals which are from Africa but were not on our list. We selected WoS, Scopus and PubMed because they represent the largest journals indexers while AJOL and AIM represent African region indexers. Additionally, WoS and Scopus are the common data sources used [21], and are criticised to be biased against journals from developing countries [22]. The search process is shown in Supplementary Figure S2 as PRISMA diagram.

### Collecting Data on Journal Metrics

We manually searched for journals metrics from Web of Science (JIF and JCI), Scopus (SNIP and Citescore), and Google scholar (H5-Index). We collected data between February and May 2023. We entered the journal metrics data in Microsoft Excel. We also downloaded bibliographic and citation data files from PubMed, WoS and Scopus to run analyses for author collaborations, and topical coverage in African health sciences journals. Unfortunately, AJOL and AIM do not provide citation data files in a format suitable for topical coverage and author collaboration analyses.

The journal metrics selected for this study are all dependent on time period; 2, 3, and 4 years and also are calculated differently hence comparison of journal impact was made within each metric and not across or against each other. The study aim was also to establish current status hence we did not compare the impact trends over a period of time. Definitions of journal metrics used in the present study include the following:

• H5-index is the number of articles (h) published in the report year and the preceding 4 years that have each been cited at least h times [23].
• The Journal Impact Factor (JIF by ISIS) is “a measure of the total number of citations for each of the papers published in that particular journal during the previous 2 years divided by the total number of eligible articles within that particular period” [24].
• The Journal Citation Indicator (JCI) is the “average Category Normalized Citation Impact (CNCI) of citable items (articles and reviews) published by a journal over a recent 3 years period” [24].
• Source Normalised Impact per Paper (SNIP) is the “ratio of the journal’s citation count per paper and the citation potential in its subject field” [25].
• Citescore is the number of citations received by “a journal in 1 year to documents published in the three previous years, divided by the number of documents indexed in Scopus published in those same 3 years” [25].

### Data Analysis and Visualisation

We used STATA version 16 [26] and Microsoft Excel version [27] to run summary descriptive statistics. We analysed topical focus coverage using VoSviewer software [28] based on all keywords co-occurrence (author keywords and MeSH keywords) mapping instead of term co-occurrence. Keywords were preferred because they define or represent the main subject matter of the article whereas terms may not necessarily represent the main subject matter of the publication or article. Co-occurrence mapping shows how many times keywords or terms appear in more than one publication. In terms of authorship collaboration, we used Vosviewer to visualise co-authorship across countries to show collaboration patterns. However, PubMed database files did not provide data on country affiliation hence we could not run co-authorship based on country names. We therefore used organisation or institutional affiliations to determine the nationality of authors for country authorship collaboration visualisations.

## Results

A total of 624 active African health sciences journals were identified, 422 from web scrapping and 202 from additional manual search. We then removed two journals whose origins were European and one Indian, hence we remained with a final list of 622 journals. However, for journal metric analyses, the study considered 221 journals because they had journal metric values collected. For country collaboration analysis, we considered 57 journals from Web of Science and 98 journals from Scopus because they could provide data in a format that would allow such analysis unlike PubMed. It is important to note that there were duplicates; some journals found in Web of Science were also indexed in Scopus or PubMed. For instance, there were 41 journals which were indexed by both Web of Science and Scopus.

### Health Science Journal Geographical Distribution Based on Indexing Status

Out of the 622 health sciences journals from Africa, 99 (15.9%) are indexed in Scopus, and 75 (12.1%) in WoS, while PubMed and AJOL list 163 (26.2%) and 172 (27.7%) journals, respectively. The African Index Medicus, which is a dedicated African region medical and health sciences journals indexer, indexes 149 (24%) of the journals. There is also an overlap where some journals are indexed by multiple databases.

**FIGURE 1:**
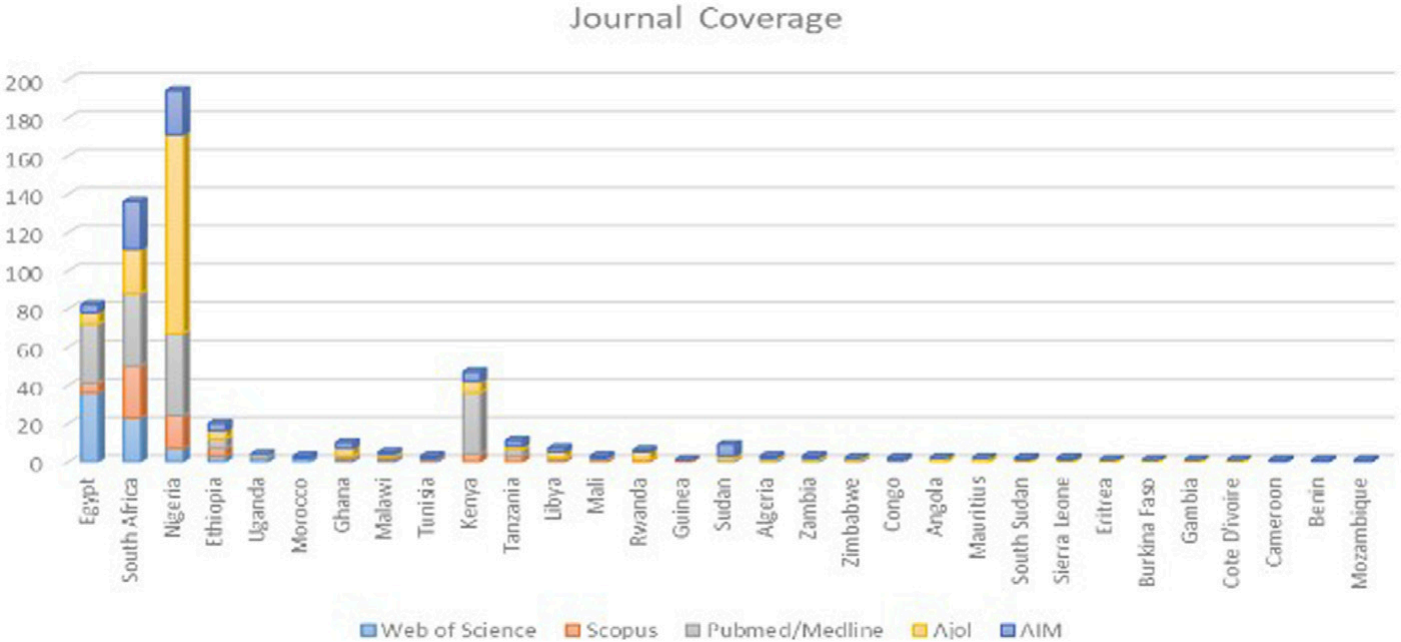
Health Science Journal geographical distribution disaggregated by indexing status (Impact of African health journals, 2023).

Considering the country in which the journals are based (Figure 1 and Supplementary Figure S3), Egypt had the highest number of journals indexed in WoS (36, 48%) while South Africa had the highest number of journals indexed in Scopus 36 (36%). Nigeria had the highest number of journals indexed in PubMed (43, 26%) and AJOL (104, 60%), while South Africa had the highest number of journals in AIM (25, 17%). About 21 African countries did not have any health sciences journal indexed in any of the international (WoS, Scopus, PubMed) and regional (AJOL, AIM) indexers under study. More than 40 countries did not have any health science journals indexed in WoS.

### Citation Scores of Journals Based on Indexing Status

#### Top Performing Journals by Country Using Interquartile Ranges

We first analysed our data using Interquartile Ranges (IQR) to show top performing journals disaggregated by country, and how the metrics of journals from different data sources are distributed per country. IQR depicts the variability of data without much influence from the outliers and skewed values. Tunisia had the highest median H5-Index with half of its journals having a median of 16 (11, 21), while Egyptian journals had highest JIF median value of 7.4 (2.1, 12.8). Similarly, Egyptian journals had the highest median values on Citescore 1.5 (0.5, 2.3), and discipline-specific metric SNIP 0.54 (0.2705, 1.1745), while South African journals had highest JCI median values 0.28 (0.16, 0.43). The countries shaded in grey had only one journal, hence had same value appearing for median, lower quartile and upper quartile. These shaded countries could not be compared with other countries with more than one journal as the median would not be statistically meaningful.

Table 1 summary statistics (Median, lower and upper quartile) of journal metrics disaggregated by country of origin. Table 1 below presents the middle 50% quartile when the metric values for journals per each country are ordered from lowest to highest. Together with the median (50%) values, are presented the lower quartile (25%) and upper quartile (75%) values in brackets Table 1 shows metrics on H5-Index, JIF and JCI, Citescore and SNIP whose data sources were Google scholar, WoS and Scopus, respectively.

**TABLE 1:** Summary statistics (Median, lower and upper quartile) of journal metrics by Country of Origin (Impact of African health journals, 2023).

Country	H5-index	JIF	JCI	Cite score	SNIP
Egypt	8 (5, 15)	7.4435 (2.087, 12.8)	0.2 (0.14, 0.49)	1.5 (0.5, 2.3)	0.54 (0.2705, 1.1745)
South Africa	9 (7, 16)	1.242 (0.982, 1.816)	0.28 (0.16, 0.43)	1.1 (0.65, 2.25)	0.532 (0.283, 0.907)
Nigeria	9 (5, 12)	0.839 (0.539, 0.961)	0.145 (0.105, 0.26)	0.6 (0.3, 1.2)	0.269 (0.1785, 0.6085)
Kenya	8.5 (4, 17)		0.42 (0.42, 0.42)	0.7 (0.5, 2.4)	0.257 (0.062, 0.471)
Uganda	30 (30, 30)	1.108 (1.108, 1.108)	0.4 (0.4, 0.4)	2.1 (2.1, 2.1)	0.581 (0.581, 0.581)
Ethiopia	10 (10, 14)	0.314 (0.128, 0.5)	0.26 (0.13, 0.39)	1 (0.8, 2.1)	0.26 (0.17, 0.35)
Libya	17 (17, 17)			2.8 (2.8, 2.8)	0.964 (0.964, 0.964)
Malawi	21 (21, 21)	1.413 (1.413, 1.413)	0.29 (0.29, 0.29)	1.6 (1.6, 1.6)	0.785 (0.785, 0.785)
Rwanda	6 (6, 6)			0.4 (0.4, 0.4)	0.147 (0.147, 0.147)
Ghana	16 (16, 16)			1 (1, 1)	0.419 (0.419, 0.419)
Morocco	4 (4, 5)				
Tanzania	13 (13, 13)			1.2 (1.2, 1.2)	0.499 (0.499, 0.499)
Zambia	6 (6, 6)				
Cameroon	15 (6, 16)			1.5 (1.5, 1.5)	0.434 (0.434, 0.434)
Benin	6 (6, 6)			0.3 (0.3, 0.3)	0.361 (0.361, 0.361)
Mali	3 (3, 3)			0.2 (0.2, 0.2)	
Tunisia	16 (11, 21)		0.36 (0.36, 0.36)	0.8 (0.8, 0.8)	0.268 (0.268, 0.268)
Sudan	7 (7, 9)				
Congo	3 (3, 3)				
Burkina Faso	2 (2, 2)				
Algeria	6 (6, 6)				
Total	9 (6, 14)	1.076 (0.6705, 1.6335)	0.26 (0.14, 0.41)	1.1 (0.5, 2)	0.471 (0.254, 0.776)

#### Top 10 and Top 20 African Health Science Journals With Highest H5-Index, JIF, Citescore, SNIP, JCI

As shown in Table 2, and Table 3, The Journal of Advanced Research from Egypt is ranked number 1 on almost all metrics regardless of data source except JCI, indicator that was not available for this journal. Thereafter, positions 2 to 10 or to 20 are occupied by different journal titles under each metric list. Also journals occupied different positions on each list, thus, a journal would be on one position on one list, and appear on another position on another list. For instance, journals such as Pan African Medical Journal and African Health Sciences have moved from positions 3 and 7 on Google scholar H5-Index to positions 39 and 32 on the SNIP list. Also, some journals (showing shaded grey boxes) do not have values for some metrics because they are not covered by the data source that was used to calculate the metrics. For some, like Scopus and WoS, had their coverage discontinued or have been newly included hence could not find recent values for metrics as the metrics are calculated for a specific period.

**TABLE 2:** Top 10 African health science journals ranked by H5-index (Impact of African health journals, 2023).

Title	Country	H5-index	SNIP	JFI	JCI	Citescore
Journal of Advanced Research	Egypt	1	1	1		1
Journal of Genetic Engineering and Biotechnology	Egypt	2	5			4
Pan African Medical Journal	Kenya^a^	3	39			21
South African Medical Journal	South Africa	4	30	2	12	9
Ethiopian Journal of Health Sciences	Ethiopia	5			11	12
Nigerian Journal of Clinical Practice	Nigeria	6	17	9	11	15
African Health Sciences	Uganda	6	32	10	10	12
Alexandria Journal of Medicine	Egypt	7			6	
Eastern Mediterranean Health Journal	Egypt	8	16	3	7	10
African Journal of Disability	South Africa	9	12		9	11
International Journal of Africa Nursing Sciences	Nigeria	9	23			15
International Journal of Veterinary Science and Medicine	Egypt	10	4		2	2
SA Journal of Industrial Psychology	South Africa	10	21		6	9

a This information is according to the data source used, however, this journal is affiliated to a number of African countries such as Uganda, Cameroon and Kenya.

**TABLE 3:** Top 20 African health sciences journals sorted by Google scholar H5-Index (Impact of African Health Journals, 2023).

Serial	Title	Country	H5-Index	SNIP	JFI	JCI	Citescore
1	Journal of Advanced Research	Egypt	60	3.548	12.8		17.1
2	Journal of Genetic Engineering and Biotechnology	Egypt	37	1.448			4.3
3	Pan African Medical Journal	Kenya^a^	37	0.509			1
4	SAMJ South African Medical Journal	South Africa	34	0.625	2.12		2.4
5	Ethiopian Journal of Health Sciences	Ethiopia	31			0.39	2.1
6	Nigerian Journal of Clinical Practice	Nigeria	30	0.802	1.12	0.39	1.6
7	African Health Sciences	Uganda	30	0.581	1.108	0.4	2.1
8	Alexandria Journal of Medicine	Egypt	28			0.5	
9	International Journal of Health Sciences	Kenya	27			0.42	2.4
10	Eastern Mediterranean Health Journal	Egypt	26	0.901	2.087	0.49	2.3
11	African Journal of Disability	South Africa	25	0.962		0.41	2.2
12	International Journal of Africa Nursing Sciences	Nigeria	25	0.743			1.6
13	International Journal of Veterinary Science and Medicine	Egypt	24	1.571		1.03	6.8
14	SA Journal of Industrial Psychology	South Africa	24	0.778		0.5	2.4
15	African Journal of Traditional, Complementary and Alternative Medicines	Nigeria	23				
16	Arab Journal of Urology	Egypt	22	1.666		0.61	5.1
17	Malawi Medical Journal	Malawi	21	0.785	1.413	0.29	1.6
18	Euro-Mediterranean Journal for Environmental Integration	Tunisia	21			0.36	
19	African Journal of Reproductive Health	Nigeria	20	0.648	0.961	0.25	1.3
20	Egyptian Journal of Medical Human Genetics	Egypt	20	0.547		0.2	1.4
21	South African Family Practice	South Africa	20	0.396		0.2	1
22	Tropical Journal of Pharmaceutical Research	Nigeria	20	0.248	0.523	0.15	0.9
23	Curationis	South Africa	19				2.3
24	ONDERSTEPOORT Journal of Veterinary Research	South Africa	18	0.921	0.982	0.76	3
25	African Journal of Emergency Medicine	South Africa	18	0.907	1.451	1.306	1.8
26	Southern African Journal of HIV Medicine	South Africa	18	0.581	1.835	0.23	2.8
27	Cardiovascular Journal of Africa	South Africa	18	0.385	0.802	0.21	1.6
28	Journal of the Egyptian Public Health Association	Egypt	17	1.646			2.3
29	Libyan Journal of Medicine	Libya	17	0.964			2.8
30	Annals of African Medicine	Nigeria	17	0.796		0.27	1.8
31	African Journal of AIDS Research	South Africa	17	0.604	1.816	0.4	2
32	Health SA Gesondheid (Online)	South Africa	17	0.532			1
33	African Journal of Food, Agriculture, Nutrition and Development	Kenya	17	0.471			0.7
34	The Nigerian Postgraduate Medical Journal	Nigeria	17				1.2
35	Journal of Applied Sciences and Environmental Management	Nigeria	17				
36	South African Journal of Psychiatry	South Africa	16	0.934	1.242	0.3	1.8
37	The Egyptian Rheumatologist	Egypt	16	0.533			2
38	The Egyptian Journal of Neurology, Psychiatry and Neurosurgery	Egypt	16	0.458			1.1
39	Ghana Medical Journal	Ghana	16	0.419			1
40	Egyptian Journal of Radiology and Nuclear Medicine	Egypt	16	0.407		0.16	0.8
41	The Egyptian Journal of Hospital Medicine	Egypt	16	0.035			
42	African Journal of Microbiology Research	Nigeria	16		0.539		
43	International Journal of Biological and chemical Sciences	Cameroon	16				
44	South African Journal of Physiotherapy	South Africa	15	1.041		0.43	1.1
45	The Egyptian Heart Journal	Egypt	15	0.663			1.4
46	Journal of Public Health in Africa	Cameroon	15	0.434			1.5
47	Egyptian Journal of Chest Diseases and Tuberculosis	Egypt	15			0.05	
48	Future of Pharmaceutical Sciences	Egypt	15				
49	Journal of the South African Veterinary Association	South Africa	14	0.78	1.044	0.57	2
50	South African Journal of Communication Disorders	South Africa	14	0.776		0.43	
51	JOURNAL OF THE EGYPTIAN NATIONAL CANCER INSTITUTE	Egypt	14	0.665		0.3	2.1
52	Ethiopian Journal of Health Development	Ethiopia	14	0.35	0.5	0.13	1
53	Annals of Medical and Health Sciences Research	Nigeria	14				

a This information is according to the data source used, however, this journal is affiliated to a number of African countries such as Uganda, Cameroon and Kenya.

In terms of countries, Egypt (15) and South Africa (15) had same number of journals, followed by Nigeria (8), on the top 20 list of journals with highest Google scholar H5-Index Table 3. Only 19 health sciences journals from Africa have their JIF calculated for 2022. Among the list, South Africa has more journals (9), followed by Nigeria (4), Egypt (2) and Ethiopia (2). Other countries like Kenya, Uganda and Malawi finish the list with one journal each.

Table 3 presents top 20 African health sciences journals sorted by Google scholar H5-Index. The table has been sorted by Google scholar H5-Index because we have more journals with Google scholar H5-Index as compared to journals with metrics calculated from WoS and Scopus data.

### Topical Focus of Journals in Different Indexing Sites

We compared topical coverage of the journals based on their indexing database. Default parameters were used for clustering which were minimum values; resolution of 1 and minimum cluster size 1. This was done to get the most studied “subjects” or “topics”. For Web of Science, 16 clusters were created with the following items in each cluster: cluster 1 (1,335), cluster 2 (1,074), cluster 3 (793), cluster 4 (748), cluster 5 (671), cluster 6 (614), cluster 7 (558), cluster 8 (466), cluster 9 (392), cluster 10 (247), cluster 11 (192), cluster 12 (167), cluster 13 (73), cluster 14 (48), cluster 15 (2), cluster 16 (1). For Scopus, nine clusters were created with the following number of items in each cluster: cluster 1 (6,277), cluster 2 (4,778), cluster 3 (2,203), cluster 4 (2,165), cluster 5 (1,978), cluster 6 (1,808), cluster 7 (1,300), cluster 8 (154), cluster 9 (21). For PubMed, nine clusters were also created with the following items under each cluster: cluster 1 (2,738), cluster 2 (2,501), cluster 3 (1,519), cluster 4 (1,031), cluster 5 (764), cluster 6 (610), cluster 7 (254), cluster 8 (67), cluster 9 (30).

**FIGURE 2:**
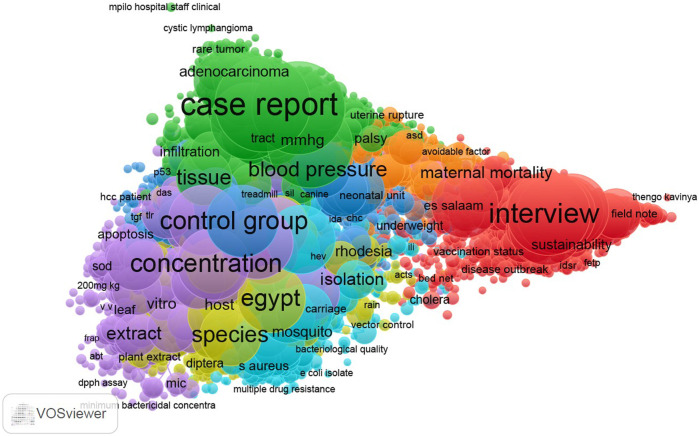
Visualisation of topical focus (PubMed Journals). It shows the most frequent keyword terms used which suggest subject focus. The size of the nodes show how frequent a keyword appeared while the colour represents clusters to which the keyword belongs to (Impact of African health journals, 2023).

The visualisation map of PubMed data (refer Figure 2) shows three types of research methods dominating which include, qualitative research, laboratory experimental studies, Randomised Control Trials and case reports. In terms of topics, infectious diseases, non-communicable diseases/chronic illnesses, maternal and child health, sexual and adolescent health, malnutrition and communicable diseases appear to be the frequently studied topics.

**FIGURE 3:**
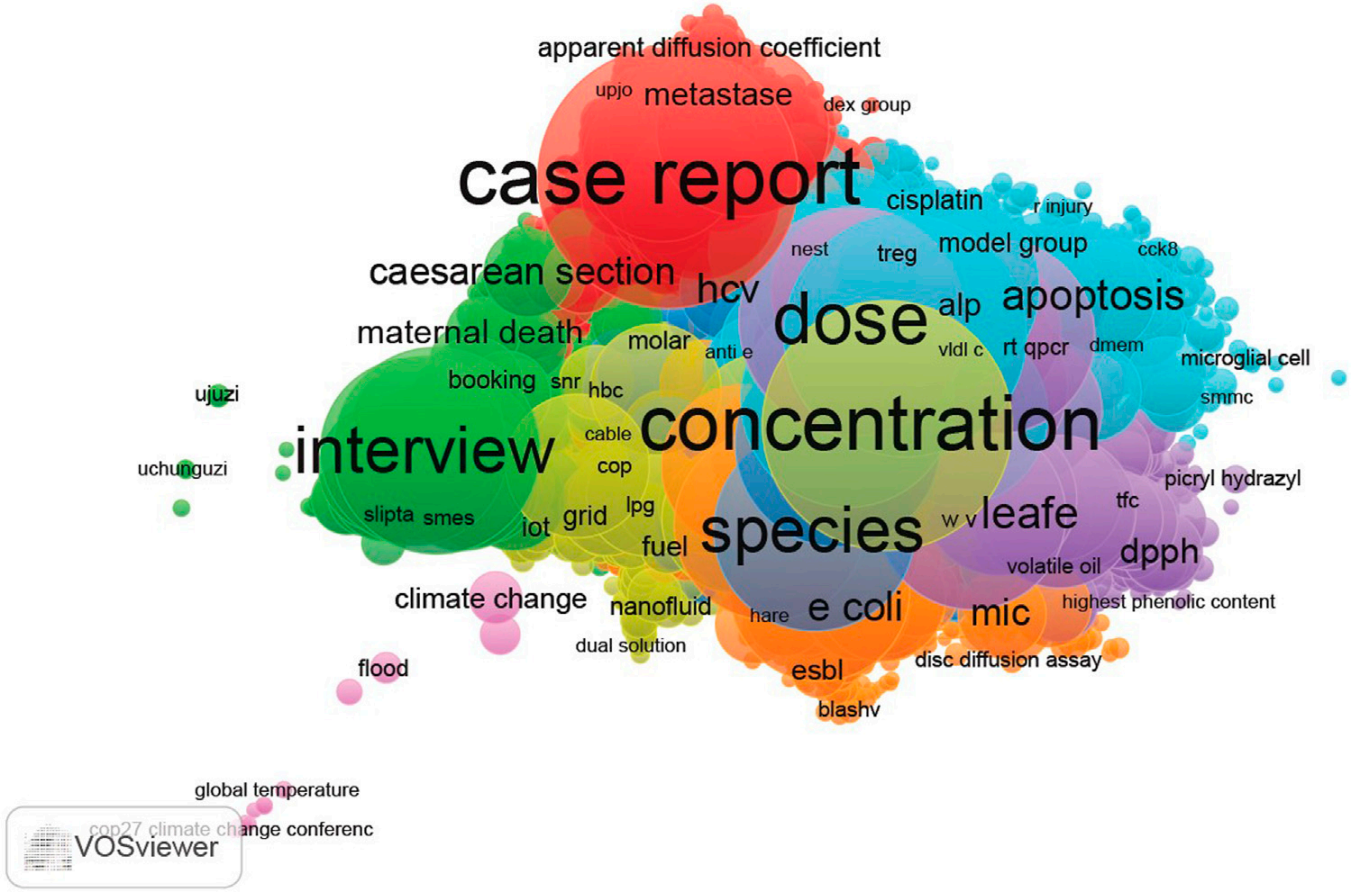
Visualisation of topical focus (Scopus journals) (Impact of African health journals, 2023).

**FIGURE 4:**
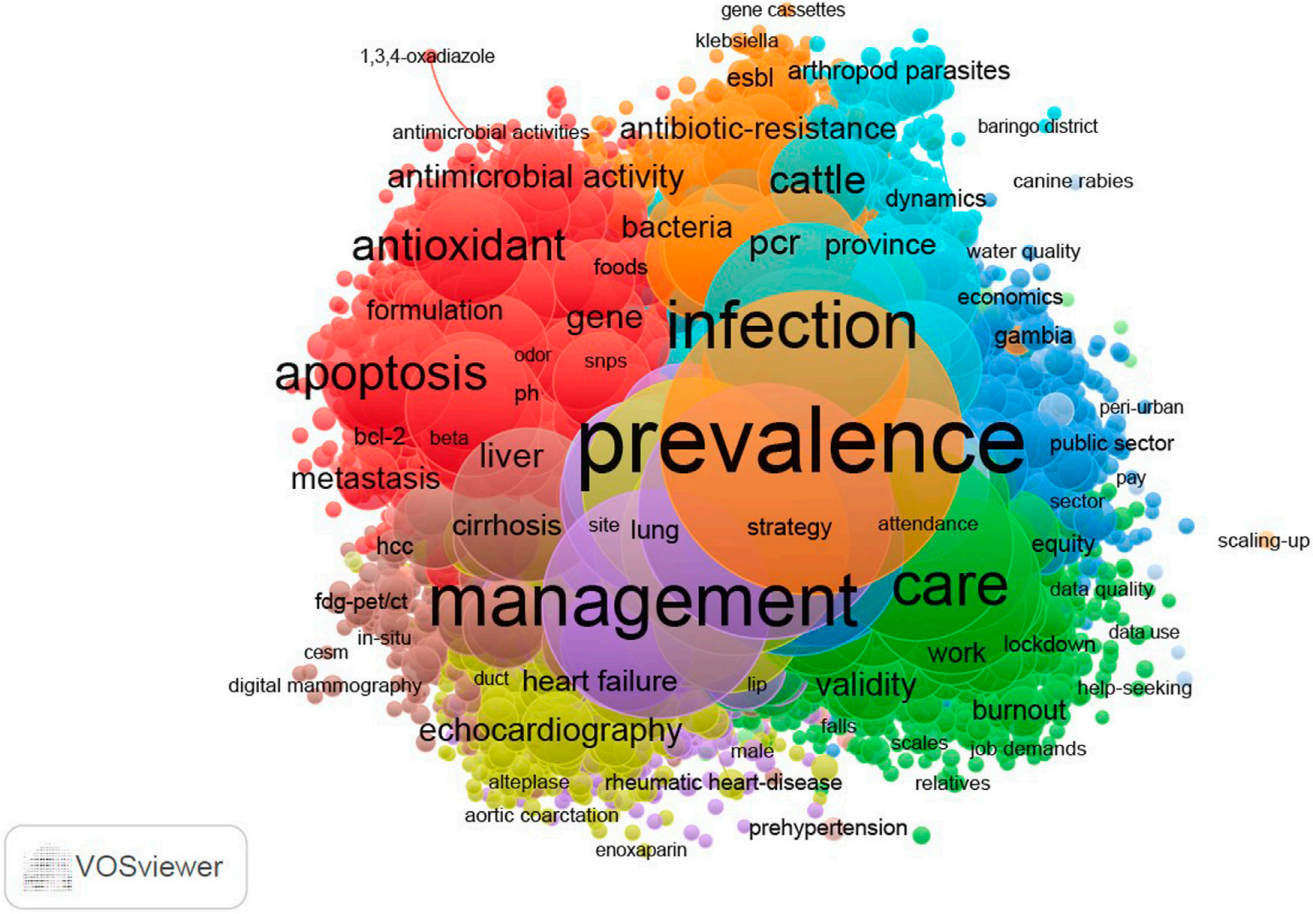
Visualisation of topical focus (Web of Science journals) (Impact of African health journals, 2023).

According to Figure 3, the visualisation map of Scopus data shows that the frequently studied topics were maternal and reproductive health, surgical conditions, physiology, chemistry and microbiology. Additionally, Figure 4 shows the visualisation map of WoS data, with three frequently studied topics which include microbiology, health systems and parasitology.

### Authorship Patterns and Collaboration of Journals in Different Indexing Sites

The network visualisations do not show observable differences in their author collaboration pattern based on database (Figures 3, 4). The results show that Egypt collaborated more with Saudi Arabia and middle east countries. In Africa, Egypt mostly collaborated with North African countries, and also with South Africa, Nigeria, Ghana, Kenya and Congo. Similarly, other North African countries such as Tunisia, Morocco, Algeria collaborate with other North African countries.

Similarly, the results suggest that countries from Southern Africa collaborated more with Southern African countries. Some countries like Lesotho and Swaziland appeared to have collaborated with South Africa and United States only. On the other hand, the data showed that Madagascar did not collaborate with any African country. Since the distance between the nodes signifies close collaborations, the maps demonstrate that Egypt is close to Middle East countries while Nigeria is very close to United Kingdom. The map shows collaboration in publications, hence availability of non-African countries and their clusters too.

## Discussion

The present study aimed to analyze the research landscape of health sciences journals in Africa. It sought to determine the coverage of African health sciences journals by international and regional indexers, examine the citation metrics of these journals based on their indexing status, visualize and compare public health topics in African health sciences journals, and assess authorship and collaboration patterns in these journals.

The study confirmed that the two major international indexers, Web of Science (WoS) and Scopus, cover less than a quarter of the total number of health sciences journals from Africa. This aligns with previous studies that cited reasons such as high editorial requirements [9], lack of capacity, and editorial bias [10, 19] for the limited inclusion of African journals in these indexes [19, 24]. However, PubMed, the major indexer for biomedical literature, indexes a majority of African health sciences journals, thus increasing their visibility and influence. Additionally, regional indexers like AJOL and AIM also cover a significant number of African health sciences journals, although the relatively low count of active indexed journals raises concerns about sustainability of local journals and visibility of local health research. This result corroborates with previous findings by Ogunfolaj et al. [18] which reported a relatively low count of 173 journals indexed by AJOL. Although a study like ours could not exhaustively review all indexing databases, the ones covered in the present study were some of the major and popular databases that offer visibility to the most important health science research around the globe [10, 19, 29]. This study still recognises that these journals may be indexed elsewhere and may still be visible and influential.

The study revealed that over half of the African countries had no health sciences journals indexed in WoS, Scopus, or PubMed, indicating limited visibility of public health science research from these countries. This lack of visibility may be attributed to factors such as the limited capacity to manage journals, journals only available in print format [1]. The dominance of countries like South Africa, Egypt, and Nigeria, which have higher research productivity and impact may be attributed to high economy and population. The present study corroborated with findings from previous studies [19] on that South Africa, Egypt and Nigeria are the most productive African countries in research, which by extension high research impact. Furthermore, the study has highlighted the limited number of African health sciences journals with Journal impact factor and Citescore, which may be due to discontinued coverage, lack of funding to meet indexing costs, or the overall struggle of African journals to secure funding [1].

The study also compared the impact of African countries based on different data sources. While Egypt, South Africa, and Nigeria consistently ranked high in traditional metrics provided by WoS and Scopus, other countries like Tunisia, Ethiopia, Kenya, and Uganda showed better performance based on Google Scholar H5-Index. This indicates that quality and not just quantity plays a role in making an impact. Furthermore, the study has revealed the discrepancies between data sources, with same journals occupying different positions on different metrics lists such as Google Scholar H5-Index compared to Scopus metrics as well as compared to JIF metrics. This underscores the importance of considering multiple data sources when assessing journal impact as there are differences in how they are calculated [10, 18, 19, 30].

Generally, results of this study have highlighted low levels of impact on different journal metrics. Since authors consider visibility and citation impact of journals when selecting a journal to publish, it implies that many local authors will consider publishing with journals with high impact from elsewhere [31] and a few African health science journals, leaving out a majority of local journals. This may limit the impact of local journals that normally focus on publishing local research that addresses local health research needs. Some authors also observed that it destroys local research as authors are forced to conduct research required by the global north journals [10].

However, the issue of measuring scientific impact is a contested one with some arguing that they cannot be relied upon due to its many inherent challenges [32]. Several other authors also suggest exercising caution when applying scientific impact metrics to measure research quality [15, 33–35]. They argue that it has a number of flaws including the different ways they are calculated, its dependence on many factors such as article types, discipline of study, source of data used to calculate metrics, and that they can also be manipulated or influenced by editorial policy. These, among other reasons, may also perhaps explain the differences in metrics in the present study.

Furthermore, the historical background of the databases may help understand its focus in terms of discipline, geographical and language coverage [30]. It is reported that Web of Science, included several journals in its collection in 2005 at the emergence of Scopus in 2004, for commercial purposes just to match the competition disregarding its previous strict quality assurance requirements [9, 36]. Some studies have even reported coverage bias towards the global north research by Web of Science and Scopus as they are originated in the global north, and significantly under-represent research from global south [22, 37]. Therefore, if one wants to judge research or calculate metrics, an understanding of data sources is essential. Considering these limitations, it is therefore important to know and consider these factors to appropriately apply the metrics to the right contexts.

Furthermore, the results of this study are also consistent with those of other studies which suggest that WoS and Scopus topical coverage lean more towards pure sciences and social sciences, respectively as compared to PubMed [9, 37]. This result can be explained by the nature of the databases. WoS and Scopus are multidisciplinary databases whereas PubMed discipline-specific databases, thus a biomedical database. The present study has shown that topical coverage for WoS and Scopus is more of natural sciences such as physics, and chemistry while PubMed has more of health-related topics such as medical conditions, public health and nursing.

In addition, previous studies have also explained that the historical nature of WoS may explain the reason it covers more natural sciences than other disciplines. For instance, Chavarro [30] asserts that WoS started with chemistry and health before humanities and social sciences journals in its collection hence has more coverage on the initial topics. He further adds that high coverage of US journals also is perhaps evidence of its origins, which is United States, and receiving support from US government indirectly at the onset of the project to compete with Russia’s scientific communication system. These differences should also be taken into account when measuring the quality and influence of a journal based on its inclusion in WoS and Scopus, as well as when using its metrics such as JIF and Citescore.

This also supports the notion of use of several data sources when assessing the visibility and impact of public health journals. For instance, this study would suggest, in addition to other multidisciplinary data sources such as Web of Science and Scopus/EMBASE, using PubMed/Medline or other discipline focused databases which have more journals in Public health. Furthermore, considering that AJOL and AIM cover a majority of African health science journals, they would be the most important data sources to base analysis on hence need for these indexers to upgrade and provide citation data for analysis.

Regarding authorship collaboration, the study found no observable differences based on the indexing database. It confirmed the pattern of collaboration based on historical colonial relationships and language, with African countries often collaborating with former colonial powers [17]. This study has established that many African countries collaborate with European countries with the exception of north African countries which have relations with the middle east. For instance, the collaboration among Anglophone countries, and also collaboration among Francophone countries. Additionally, even research funding or assistance is given to African countries based on former colonial relationships. This creates power imbalances in research agendas, where African countries are underrepresented and research priorities are influenced by the global north [38]. The findings of this study also agree with a study by Hernandez-Villafuerte [39] on that there is less collaboration among authors from African countries.

### Study Limitations

The study acknowledged its limitations, including the possibility of missing some African health sciences journals not indexed in the searched data sources. Other analyses which did not use data from AJOL and AIM may also under represent the scientific impact of the journals from Africa. It also focused on journals with online presence only, excluding print-only journals.

### Conclusion

Visibility of African health science journals is still a challenge that needs to be addressed to increase access to Africa’s health science research, and its impact on public health policy and practice. The low coverage, including complete absence of health sciences journals by some African countries perhaps point towards inequalities in our health systems that require comprehensive and sustainable solutions. It has also revealed gaps in regional indexers, which cannot provide citation data for analysis despite indexing majority of African health science journals. In addition, the findings of this study have added evidence and significance to the assertions that several data sources and metrics need to be consulted when assessing journal quality and research impact. This also applies to a researcher, institution and country’s research productivity and impact. Caution should be exercised in applying research metrics, considering contextual factors and limitations such as purpose for doing assessment, choice of appropriate metrics, interpretation and also data sources used. The purpose for doing assessment will determine the appropriate metrics to use and data sources to consult based on contextual factors such as region, language, discipline and countries level of development. It is also important to highlight limitations together with the presentation of the results or metrics for contextual understanding and appropriate application to policy and practice.

### Implication for Research

These findings call for further research to explore barriers and facilitators for indexing African health sciences journals and increasing collaboration between African countries.

### Implication for Policy and Practice

This study emphasizes the importance of understanding and applying research metrics appropriately in policy and practice, considering contextual factors and exercising caution. Researchers need to consult several research databases for their research needs and answers such as selection of journal to publish and research evidence for practice. Discipline of study also needs to be considered when choosing which databases to consult when searching for research evidence. When formulating research evaluation policies such as promotions and tenure decisions in universities, there is need for proper understanding of research impact assessment, and also several contextual factors need to be considered and applied with caution.

## Supplementary Material

Supplementary Figure S1 Scientometric analysis process (Impact of African health journals, 2023).

Supplementary Figure S2 Search process PRISMA flow diagram (Impact of African Health Journals, 2023).

Supplementary Figure S3 Map of Africa showing journal coverage for each database by country (Impact of African health journals, 2023).
