# Supplementary figures and images for: A Scientometric Analysis of Africa’s Health Science Journals Indexed in International and Regional Databases: A Comparative Analysis
Source: Int J Public Health. 2024 Jan 15;68:1606415. doi: 10.3389/ijph.2023.1606415 (PMC10851738; doi:10.3389/ijph.2023.1606415)

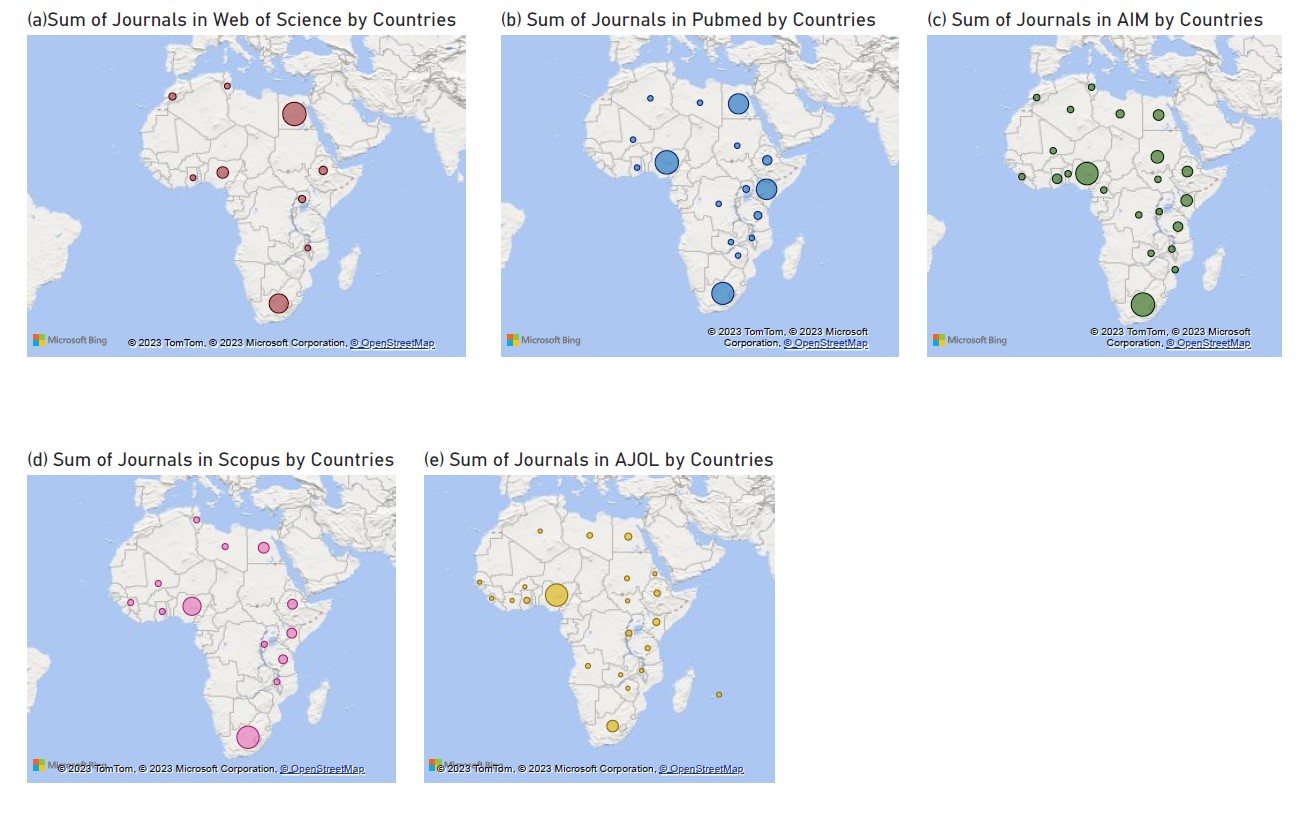

Supplement: Supplementary file 1 [file Image3.jpg]

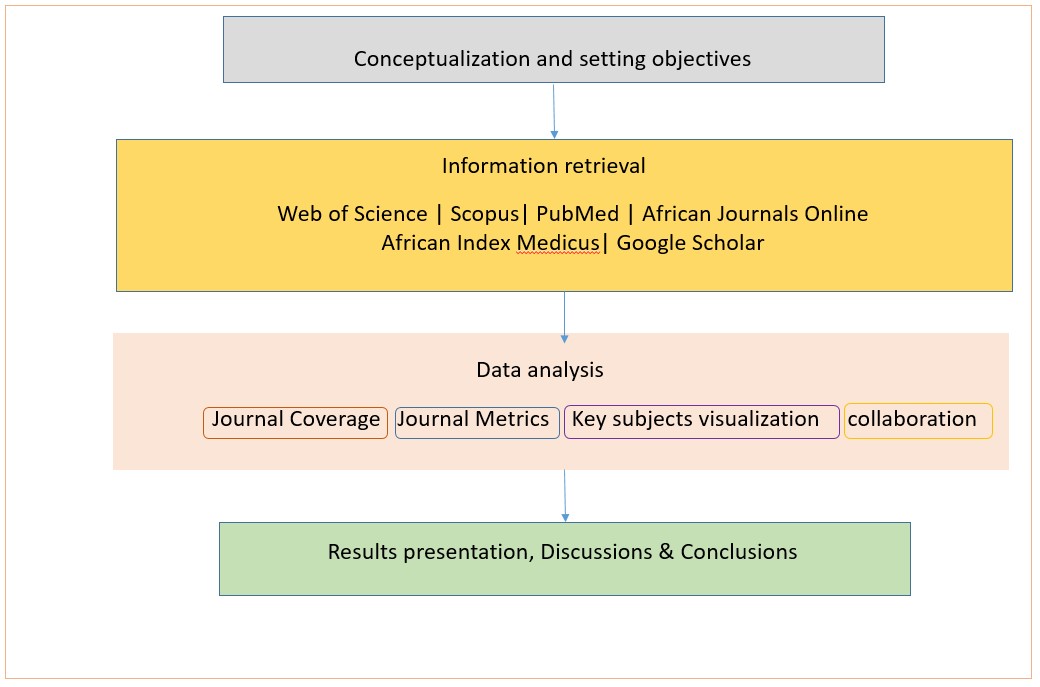

Supplement: Supplementary file 2 [file Image1.jpeg]

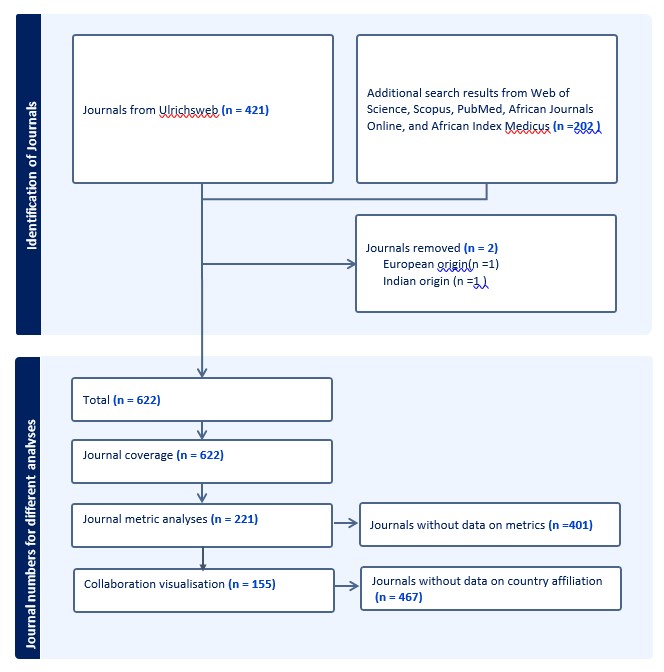

Supplement: Supplementary file 3 [file Image2.jpeg]
